# Label-free methods for optical *in vitro* characterization of protein–protein interactions

**DOI:** 10.1039/d1cp01072g

**Published:** 2021-08-03

**Authors:** Fabian Soltermann, Weston B. Struwe, Philipp Kukura

**Affiliations:** Physical and Theoretical Chemistry, Department of Chemistry, University of Oxford UK philipp.kukura@chem.ox.ac.uk

## Abstract

Protein–protein interactions are involved in the regulation and function of the majority of cellular processes. As a result, much effort has been aimed at the development of methodologies capable of quantifying protein–protein interactions, with label-free methods being of particular interest due to the associated simplified workflows and minimisation of label-induced perturbations. Here, we review recent advances in optical technologies providing label-free *in vitro* measurements of affinities and kinetics. We provide an overview and comparison of existing techniques and their principles, discussing advantages, limitations, and recent applications.

## Abstract 2

Protein–protein interactions are involved in the regulation and function of the majority of cellular processes.

## Graphical abstract

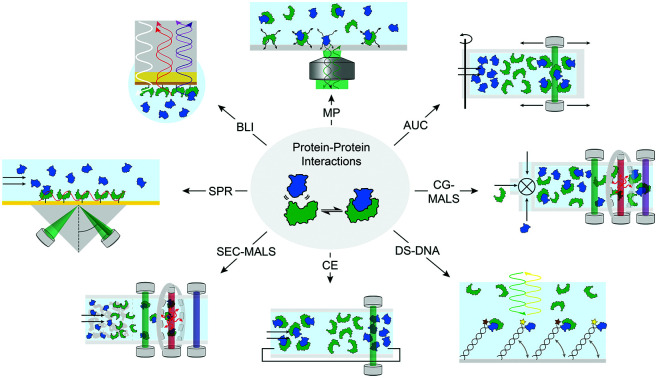

## Introduction

It is estimated that the human genome codes for more than 500 000 different proteins, of which cells can produce more than 10 000 at any given time.^1^ Given the vital role of proteins in the highly regulated environment of our body, it is not surprising that about 80% of all proteins are expected to function in cooperation with other proteins and substances.^1^ Therefore, proteins have to be viewed as part of a complex network of interactions, where changes of one part can induce a cascade of changes in another. Identifying and understanding individual protein interactions helps to break down the network into more manageable pieces, enabling us to reveal these complex mechanisms with the ultimate goal of understanding the basis of malfunction and identifying and optimizing routes to intervention. In this perspective, we discuss some of the most commonly used optical methods applied to quantify protein–protein interactions *in vitro*.

Before interactions can be quantified *in vitro*, interacting proteins of interest have to be identified, ideally under conditions as close as possible to *in vivo.* Two commonly used approaches to screen for multi-protein complexes *in vivo* involve affinity tags and two-hybrid screens. In affinity-tag methods, the protein of interest is expressed with a genetically fused affinity tag (*e.g.* 6xHis) for purification.^2^ Putative binding partners are expected to bind to the affinity-tagged protein *in vivo*. During purification, the tagged protein (bait protein), together with bound proteins, is captured with ligands linked to a solid support (*e.g.* Ni–NTA resin). After washing away the cell lysate (and all non-interacting proteins), the bait protein with its bound proteins is eluted and identified by mass spectrometry.^3^ With advances in mass spectrometry instrumentation, especially in quantitative proteomics and native mass spectrometry, the sensitivity of detection protocols has significantly improved. Although the affinity-tag method allows for high throughput, it is biased towards high-affinity interactions and slow kinetics. Other commonly used methods include co-immunoprecipitation and cross-linking.^4^

More laborious, but also more reliable, are two-hybrid screens, *e.g.* the yeast two-hybrid system.^5^ In this method, the protein of interest is fused to a DNA-binding domain during expression. Similarly, its putative binding partner is fused to a transcription activation domain. If the putative binding partner binds to the protein of interest this activates a downstream reporter gene.^4^ This reporter can be either an auxotrophic or colorimetric reporter making this method suitable for high throughput screening. It is estimated that 30–60% false positive and 40–80% false negatives are obtained in high-throughput studies that use two-hybrid or affinity-based techniques.^1^ Therefore, it is important to increase accuracy by combining several techniques.

Once putative binding partners are identified, they are often recombinantly expressed and purified to obtain sufficient sample for an in-depth characterization of the proteins and their interactions. In this process, purity, integrity, and activity are iteratively checked with methods including chromatography (*e.g.* SEC), SDS–PAGE or ELISA. When the desired quality criteria are met, the proteins and their interactions can be characterized with a range of *in vitro* biochemical and biophysical techniques.^6^ Compared to *in vivo* methods, investigating protein interactions *in vitro* reduces complexity and simplifies data interpretation. With increased throughput, it also makes it possible to efficiently test different mutations, ligands and conditions.

**Fig. 1:**
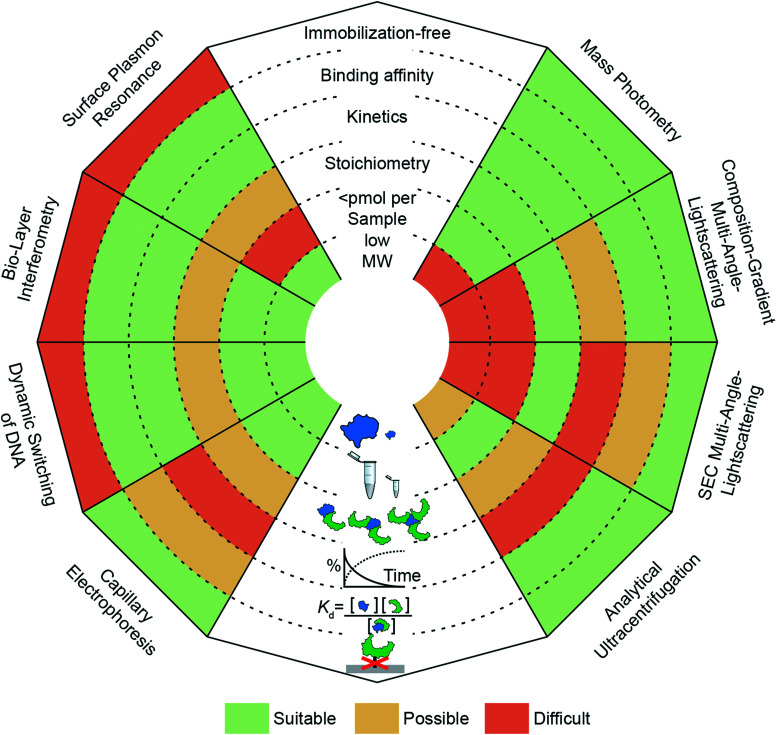
Overview of optical methods for protein–protein interaction quantification *in vitro*. SPR, BLI, dynamic switching of DNA layers, CE, AUC, SEC-MALS, CG-MALS and mass photometry are discussed (anti-clockwise from top left to top right) and separated into three categories (“surface-based”, “separation-based”, “solution-based”). Each “ray” of the spider-diagram represents one method and is divided into a set of criteria (immobilization-free; being able to measure binding affinity, kinetics, stoichiometry, <pmol per sample, low molecular weight species). The methods’ performance for each criterion are compared by a color code. This code indicates to which degree a method meets a criterion. “Suitable” (green), “Possible” (yellow) and “Difficult” (orange).

The need to quantify protein–protein interactions arises frequently in all areas of the life sciences and while a range of different techniques are available to do so, it remains a challenging task. In particular, going beyond a simple yes/no assessment to confidently quantify bound and unbound states, as well as small changes in their abundance, requires high levels of sensitivity and reproducibility. In this regard, label-free, optical methods are advantageous because they are non-invasive, access a wide concentration range, require low sample amounts and have the potential for high-throughput analysis. In Fig. 1, we present an overview of widely applied label-free optical methods for quantification of protein–protein interactions *in vitro*, which are covered in this perspective. Other techniques, such as native mass spectrometry,^7^ isothermal calorimetry,^8^ protein charge transfer spectra^9^ or fluorescence-based techniques,^10–14^ are beyond the scope of this perspective and are discussed elsewhere.

Label-free or immobilization-free techniques are generally highly sought after because labelling or immobilizing analytes/ligands can alter the measured binding interaction^15^ and requires an additional step in the experimental procedure.

The ability of a technique to access a wide concentration range goes along with a large dynamic range for *K*_d_ measurements, *i.e.* binding affinity measurements. In other words, weak interactions require high concentrations to contain a detectable amount of complex and usually exhibit fast off-rates, which makes them difficult to capture at concentrations below the *K*_d_ value. In contrast, strong interactions (sub-μM) require high sensitivity to quantify the low abundance of unbound species or measurements at concentrations within an order of magnitude of the *K*_d_ value, which can be nM or below.

Kinetic measurements can be seen as a more elaborate version of binding affinity measurements because they involve quantifying the abundance of bound and unbound species at different time points, whereas binding affinity measurements take place after equilibration. In kinetic measurements, two compounds are mixed to observe complex formation, or an equilibrated mixture is diluted to observe complex dissociation. In both cases, on- and off-rates need to be taken into account to reveal the association or dissociation kinetics. The accessible on- and off-rates can cover time scales from milli-seconds to hours or even days. Following reactions with high sensitivity over long periods of time is demanding because noise and baselines must be reproducible or at least corrected for.

An additional layer of complexity is added when working with multi-valent or oligomeric proteins, *i.e.* molecules with more than one binding site. Here, bound and unbound states can consist of complexes with different stoichiometries. Interpretation of this binding data often requires fitting procedures where different binding models are compared to the experimental data to deduce the stoichiometry. Techniques that can measure molecular weights offer an advantage here, because they can directly identify oligomeric states and complexes. In this regard, molecular weight resolution as well as dynamic range of molecular weight detection are important factors to consider.

Finally, a parameter whose importance is often underestimated when selecting a method is the amount of sample required to perform the experiment. Sample availability can be limited due to several factors, such as low expression levels or low recovery after purification.^16^ Although, this is in principle only a technical problem, in practice, preparing larger amounts of sample can be costly and time-consuming. This issue has encouraged the development of miniaturized or chip-based methods, which can reduce sample amounts to sub-μM concentrations with only μl of sample.^17^ Beyond these criteria, further factors to consider when selecting a method are its versatility (*e.g.* applicability to different classes of biomolecules, buffers or complex samples such as cell-lysates), degree of automation, and throughput.

We emphasize that this perspective is not intended to be a comprehensive overview of the field as a whole. Instead, we aim to present recent advances to illustrate the capabilities of different analytical techniques using light in the broader sense, capable of quantifying protein–protein interactions without the need for labels.

## Immobilization-based methods

### Surface plasmon resonance (SPR)

**Fig. 2:**
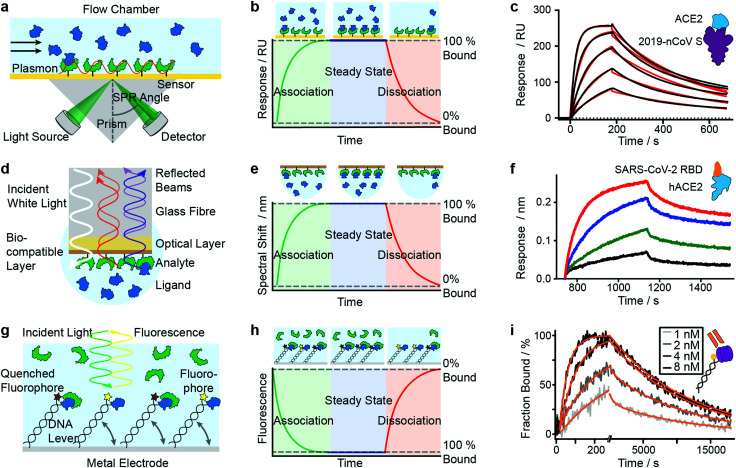
Immobilization-based methods for quantifying protein–protein (PPI) interactions. Principles and experimental data of surface plasmon resonance (a–c), bio-layer interferometry (d–f) and dynamic switching of DNA layers (g–i). Analyte (green) or ligand (blue) are immobilized onto a surface and then exposed to a solution containing ligand or analyte of known concentration. The formation of complex can then be followed with time to extract on- and off-rates of interactions as well as the binding affinity. Adapted from ref. 22 with permission from AAAS (c). Adapted from ref. 23 with permission from Springer Nature (f). Adapted from ref. 24 with permission from Springer Nature (i).

SPR has seen considerable growth in popularity since its commercialization in the 1990s,^18–20^ providing label-free quantification of protein–ligand affinities and kinetics.^1,21^ In an SPR experiment, the analyte (or ligand) is immobilized to a sensor surface—usually a thin gold layer on a glass support (Fig. 2a). The sensor surface is then illuminated with polarized light at an angle that excites surface plasmons, known as the SPR angle. Small changes in the refractive index of the sensor surface (*e.g.* caused by molecules binding to the surface) will affect the SPR angle and the detected light intensity, which is reported in response units (RU). Therefore, RUs are proportional to the amount of ligand bound to the analyte on the sensor surface.

After the immobilization step, the analyte is exposed to a continuous flow of ligand. Ligand molecules binding to the analyte will change the SPR angle and lead to an increase in RU, yielding an association curve (Fig. 2b). Once the RUs are constant, either all binding sites on the surface are saturated or the system has reached chemical equilibrium. At this point, the ligand solution is replaced by blank buffer, which makes dissociation the dominating process. This results in a decrease in RUs, yielding a dissociation curve. Both association and dissociation curves are characterized by the on- and off-rates of the interaction. Knowing the concentration of molecules in solution and fitting the binding curves, based on the Langmuir isotherm, provides *k*_on_ and *k*_off_ and consequently also the *K*_d_. Although different binding models are available, recent literature mostly relies on 1 : 1 interaction models, implying that SPR is mostly used to characterize simple stoichiometries.

The strengths of SPR are its large dynamic range for *K*_d_ measurements (sub-nM to low mM) and the small sample amounts required (several μg per sensor chip).^6^ Experimental conditions are compatible with different buffers, although care needs to be taken when using detergents, chelating agents or denaturing agents. Measurements are fully automated and the sample preparation steps needed are minimal.

Despite the high degree of automation, SPR remains a mostly low-throughput method because several runs are needed to obtain robust measurement of kinetics and binding affinities, which can take several hours. Further limiting factors are the requirement that the ligand be immobilized to the sensor surface;^6^ mass transport effects, which limit the upper limit of accessible kinetic processes (*k*_off_ < 10^−1^ s^−1^); and sensitivity of SPR to non-specific interactions between sensor surface and analyte.^25^

Despite these limitations, SPR is extremely popular due to being user-friendly and having broad applicability to various biomolecule classes. Given the large number of SPR publications, we refer here to reviews showcasing recent developments in SPR applications for protein–protein interaction quantification,^26–28^ high-throughput with SPR imaging sensors (SPRi),^29^ sensitivity and detection speed,^30,31^ influence of capture surfaces,^32^ and overcoming challenges with multi-valent binding.^33,34^

Mamer *et al.*, in their review, show SPR's convergence to cell-based protein–protein interaction measurements.^35^ Recent research focusing on SARS-CoV-2 protein interactions highlights the applicability of SPR, as shown in Fig. 2c, where Wrapp *et al.* quantified the affinity of the SARS-CoV-2 spike protein (violet) binding to neck-domain-free ACE2 (blue).^22^ His-tagged S protein was immobilized to a Ni–NTA sensorchip and exposed to serial dilutions of untagged ACE2 (250 to 15.6 nM), each yielding a sensorgram (black lines). The experimental data was fitted with a 1 : 1 Langmuir model (red), revealing binding kinetics of *k*_off_ = 2.76 × 10^−3^ s^−1^ and *k*_on_ = 1.88 × 10^5^ M^−1^ s^−1^, and a *K*_d_ of 14.7 nM. Future work is directed towards increasing SPR's sensitivity and specificity—which will further expand its applicability to various classes of biomolecules and their interactions.

### Bio-layer interferometry (BLI)

BLI is an efficient tool for characterizing interactions between various classes of biomolecules and is often seen as the high-throughput alternative to SPR. Commercially introduced 15 years ago its popularity as a biosensor technology grew rapidly. It makes it possible to determine kinetic rate constants and the binding affinities of molecular interactions, without the need for labels.^36^ BLI is similar to SPR in the sense that both require immobilization of a ligand on a sensor surface, where analyte binding is detected using an all-optical method (Fig. 2d). In BLI, white light is directed through an optical fiber to a biocompatible layer on the fiber surface. Once ligand molecules are immobilized on this biolayer, the fiber becomes a probe which can be dipped into analyte solution.

Analyte binding changes the refractive index of the biocompatible layer, which is measured as a change in interference pattern and is proportional to the amount of bound analyte. The interference arises from the small path difference between light reflected from the fiber surface/biolayer interface and the biolayer/solution interface.^37^

In contrast to SPR, where the binding surface is exposed to a continuous flow, the BLI sensor tip is dipped in static solutions of ligand and repeated for a series of dilutions. The use of orbital agitation of the sample holder at high speed ensures sufficient mixing to minimize mass transport limitations. With this approach, association and dissociation of the analyte and binding partner can be followed over time to extract *k*_on_ and *k*_off_ (Fig. 2e) and binding affinities in the range of 10 pM to 1 mM are accessible. In many cases, a 1 : 1 interaction model is applied to fit the model to experimental data.^38,39^

Given that experiments can be performed in micro-well plates, no maintenance-intensive microfluidics are needed. Setups can be combined with disposable sensor tips, which enables coupling to 96- or even 384-well high-throughput formats.^40,41^ However, the advantage of higher-throughput comes with inferior reproducibility and lower sensitivity to low MW analytes, so cross-validation by SPR is generally recommended.^40^

Similar to SPR, BLI has limitations due to mass transfer and immobilization effects.^36^ A discussion of immobilization effects can be found in the work of Kamat *et al.* where they developed a binding kinetic assay to quantify antigen–antibody interactions.^42^ Advances in data analysis should further help to go beyond the often assumed 1 : 1 interaction models.^43,44^

Among recent applications since 2019 are several studies focusing on SARS-CoV and MERS-CoV interactions. In Fig. 2f, Yi *et al.* use BLI to compare binding affinities of full-length human ACE2 (blue) to the receptor binding domain (RBD) of SARS-CoV-2 (orange).^23^ Data was recorded on an Octet RED96 instrument at different concentrations (1.85 nM (black), 5.56 nM (green), 16.67 nM (blue), 50 nM (red)). Binding kinetics were evaluated with a 1 : 1 Langmuir binding model by ForteBio Data Analysis 9.0 software and yielded *k*_on_ = 2.65 × 10^5^ M^−1^ s^−1^, *k*_off_ = 1.35 × 10^−3^ s^−1^ and *K*_d_ = 5.1 nM.

In similar studies, BLI has been used to study the spike glycoprotein (HIV-1, SARS-CoV-2, MERS-CoV) and its binding to receptors and antibodies,^22,45–49^ including antibody competition assays for humoral protection.^50,51^ BLI has also been used to quantify cross-reactivity of antibodies for Nipah virus (NiV) and Hendra virus (HeV), against which no vaccines or licensed therapeutics exist yet.^52^ Li *et al.* made use of the concentration-dependent on-rates of antigens binding to immobilized antibodies to quantify antigen levels in a high-throughput manner in CHO cell line development. They compared BLI's sensitivity and throughput to widely-used assay formats, such as ELISA.^53^ A similar approach was used by Wallner *et al.* to quantify glycosylation of Fc-glycosylated IgGs *via* immobilized lectins as measure for product quality.^54^ Loomis and Steward-Jones *et al.* used BLI to evaluate the antigenicity of their candidate vaccines in structure-based design of Nipha virus vaccines.^55^

In summary, BLI is widely used for binding affinity and kinetics measurements of protein–protein interactions, and is increasingly applied as a complementary method to SPR.

### Dynamic switching of DNA layers

The use of DNA layers has emerged within the past 10 years and is used to obtain protein interaction parameters (*i.e.* affinity, kinetics) and additional information such as size, shape, flexibility and elasticity of the protein complexes, with quantities of less than 10^−18^ mol on the sensor surface.^56,57^ The principle is similar to BLI and SPR in that it uses ligands immobilized on a surface to capture analyte molecules. A fluorescent marker (*e.g.* Cy3 dye) is attached to a double-stranded DNA oligonucleotide, which is tethered to a gold surface (Fig. 2g). A ligand (*e.g.* an antigen) is then immobilized at the solution-exposed end of the DNA tether, in proximity to the fluorescent marker. Binding of the analyte to the ligand quenches the fluorescence and is detected as a change in fluorescence signal. Exposing the immobilized ligand to a continuous flow of analyte solution leads to a continuous decrease in fluorescence intensity (association curve) until all ligand molecules are saturated or a steady-state is reached in which the fluorescence signal reaches its minimum (Fig. 2h). Switching to a blank buffer solution leads to dissociation of analytes until the initial fluorescence signal is restored. Fitting kinetic models to the experimental data yields on- and off-rates as well as binding affinities.

Alternatively, experiments can be configured to use the DNA tether as a sensitive indicator for size, shape, flexibility and elasticity of the protein complexes.^56^ The negatively charged DNA tether can be induced to switch between horizontal and vertical conformations by alternating electric fields (a few 100 mV in the kHz range, with spatial extension of only a few nanometers). These fields are applied to the gold surface covered with a low-density double-stranded DNA monolayer. In the “standing” position, fluorescence is detectable, whereas it is quenched in the “lying” conformation by nonradiative energy transfer from the dye to surface plasmons in the gold surface. The type of charged polymer (*e.g.* DNA), the grafting density, and the composition and ionic strength of the buffer solution play important roles in the switching behavior. Parameters such as charge, size, shape, conformation, flexibility and internal elasticity alter the switching behavior.^58^ Together with changes in fluorescence properties due to the proximity of the marker and bait protein, these parameters influence the fluorescence signal.^57^ Their contribution must be carefully dissected when interpreting the signal. For further discussion of these experiments we refer readers to the work of Rant *et al.*^57^ The approach can also be used for interactions involving small-molecules, or DNA/RNA interactions.^59^ We do not discuss that further here but instead refer readers to the review of Rant *et al.*^57^

Early binding affinity experiments demonstrated that changes in switching amplitudes can be detected when exposing immobilized sheep IgG to a protein G solution.^56^ This work also showed that the sensitivity was sufficient to work in the low fM concentration range with sample quantities of bound protein as little as 0.3 amol, compared to a minimum of 20 amol for SPR.^56^

A more recent measurement of binding affinities and kinetics investigated interactions between strawberry fruit allergens (Fra a 1.01E, Fra a 1.02 and Fra a 1.03) and the putative binding partner FaAP, which was identified with the help of a yeast two-hybrid screen and sequence matching.^60^ The interaction study confirmed the putative binding and revealed nM affinities for all systems, with rate constants varying by 2 orders of magnitude.

In 2018, Crowe *et al.* applied this technique in the preclinical development of a novel, orally administered anti-TNF-α domain antibody for the treatment of inflammatory bowel disease. Both their antibody candidate, V565, and the clinical comparator, antibody adalimumab, showed picomolar affinity for human sTNF-α.^61^

In a further study by Daub *et al.* in 2020, the stability of intrinsic multi-valent complexes formed by TNF-α trimers bound to TNF-α-scavengers (IgGs and Fab fragments) was investigated.^24^ The real-time assessment of TNFα monomerization helped to improve the understanding of TNFα's bioactivity and its role in regulating inflammation. In Fig. 2i, immobilized TNF-α (violet) was exposed to Adalimumab Fab (orange). Solutions of Adalimumab Fab diluted in running buffer (1, 2, 4, 8 nM) were injected for 260 s (association), followed by injection of running buffer for 18 000 s (dissociation). Solid light grey to black lines represent normalized data. Solid orange lines represent global fit data. Kinetic rates and affinities were as follows: *k*_on_ = 2.82 × 10^6^ M^−1^ s^−1^, *k*_off_ = 8.52 × 10^−5^ s^−1^ and *K*_d_ = 30.2 pM.

These experiments show that switching DNA layers can provide insights comparable to those that can be obtained using SPR and BLI. Although still a relatively new method, it offers new avenues to accelerate interaction measurements because it is chip-based, and could be parallelized and adapted to high-throughput microarrays.^58^

## Separation-based methods

### Capillary electrophoresis (CE)

**Fig. 3:**
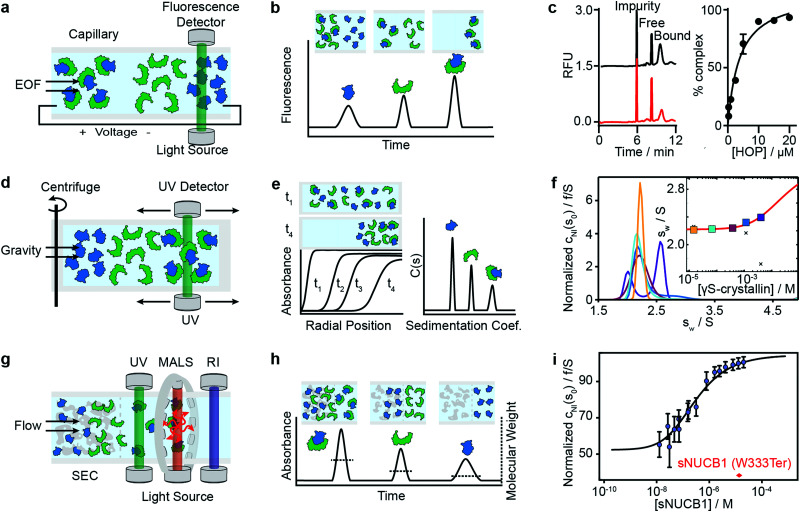
Separation-based methods for PPI quantification. Principles and experimental data of capillary electrophoresis (a–c), analytical ultracentrifugation (d–f) and size-exclusion-chromatography multi-angle light scattering (g–i) are shown. Analyte (green), ligand (blue) and complex are separated while total concentration is monitored with UV or fluorescence. In MALS a refractive index detector and multi-angle light scattering detector are added to determine the average molecular weight of species present in solution. The abundance of unbound and bound species can be either determined directly from the peak areas or *via* modelling and deconvolution of peak shapes. Adapted from ref. 65 with permission of RSC (c). Adapted from ref. 66 with permission of ACS (f). Adapted from ref. 67 with permission of ASBMB (i).

Capillary electrophoresis separates proteins based on differences in migration velocity determined by their mass and charge in a separation channel under an applied electric field (Fig. 3a). Relative abundances of separated proteins are typically detected after elution with mass spectrometry or optical methods such as UV absorbance or laser-induced fluorescence.^62^ CE has become a powerful screening tool in drug discovery using the presence or absence of peaks in the elution profile as an indicator for the formation of interactions (Fig. 3b). It enables large-scale inhibition assays of protein interactions with small molecule drugs with minimal sample consumption (nanoliters or less), low sample requirements, ease of automation, high throughput and fast measurement times (below 1 s were achieved).^63^ A microfluidic version of CE (MCE), has been commercialized for interaction screening.^64^

A limitation of CE is that it is highly sensitive to changes in buffer composition because such changes directly influence the electroosmotic flow and mobility of proteins and therefore the elution time and resolution. Moreover, for certain applications, more sensitive fluorescence detection may be required because of low concentrations or thin channel diameters, such as in microfluidic devices.^68^

A selection of studies, however, highlight the applicability of CE for measurements of protein–protein binding affinities. Recently, Rauch *et al.* quantified the affinity of labeled Hsp70 binding to Bag3 for a 1 : 1 binding model, obtaining a *K*_d_ of 23 ± 8 nM.^69^ Their comparison of CE results with SPR and ITC measurements showed excellent agreement. They also showed, in a competition experiment, that adding unlabeled Hsp70 led to a decrease in the complex and resulted in an IC_50_ of 0.24 μM, highlighting the suitability of CE for inhibition assays.

Earlier examples include application of CE to determine the *K*_d_ for Gc-globulin G-actin, lectin–glycoprotein and antibody–antigen interactions.^70,71^ Various antibody–antigen binding affinities and stoichiometries have been investigated by CE-methods and upon comparison with standard methods shown to be accurate and precise.^31,63^ A good summary thereof can be found in the review of Moser *et al.*^63^

In another example, Lassen *et al.*^72^ could not determine a valid binding constant for the C4–mAb interaction using CE and attributed this to various factors, such as C4 adsorbing to the inner wall of the capillary, which decreased the electroendosmotic flow at high C4-concentrations, and to the multi-valency of the interaction.

Besides the general issue of protein adsorption to the inner wall of the capillary,^14,64,73^ the difficulty of maintaining protein complexes while still allowing separation with sufficient resolution poses a major limitation.^73^ However, this can be addressed with protein cross-linking. Cross-linked proteins can be exposed to harsher conditions and longer separation times while still maintaining the initial interaction.

Ouimet *et al.* used protein cross-linking electrophoresis (PXCE) to determine the *K*_d_ of various interactions, which were all in agreement with other methods, *e.g.* lysozyme–antilysozyme (24 ± 3 nM, compared to 17 ± 2 nM from ITC) immunocomplex, Hsp70–Bag3 heterodimer (25 ± 5 nM, compared to 23 ± 8 nM from CE), and Hsp90 homodimers (2.6 ± 0.3 nM, compared to 60 ± 12 nM from size exclusion chromatography).^74^

The same group further developed this method with an accelerated cross-linking method, which increased throughput to 1 min per sample and showed its validity using 5 different protein–protein interactions.^65^ For the Hsp70–HOP interaction, they obtained an electropherogram (left) and saturation binding curve (right) by 10 s glutaraldehyde cross-linking (Fig. 3c). Saturation binding curves were fit by nonlinear regression to find a *K*_d_ = 3.8 ± 0.7 μM. With their method, they were able to separate impurities from the proteins of interest and quantify the abundance of bound and unbound states. Their data shows that even low-affinity interactions can be quantified using PXCE and that its sensitivity is sufficient for use in competition or inhibition assays.^73^

Further advances have been made to make CE more suitable for the screening and discovery of PPI inhibitors, namely coupling of chemical cross-linking methods to CE for high-throughput screening of cell lysates^75^ and coupling to 96-well plates.^69^ Recent, commercially available CE systems have been extended to multiple channels and can also be coupled to high throughput formats.^64^ Ouimet *et al.* showed that they can further increase throughput with a novel droplet sample introduction system which interfaces the multi-well plate and microchip gel electrophoresis more efficiently, resulting in overall run times of 10 s per sample.^76^ These kinds of microfluidic approaches combined with high-throughput compatibility, *i.e.* coupling to plate readers and robotic automation, will pave the way for broader application.^11^

Although recent CE literature reporting binding affinity measurements of protein–protein interactions is limited, CE's potential lies in its ability to deliver yes/no answers quickly, which makes it a powerful method for high-throughput screening of small molecule inhibitors of interactions under physiological conditions.^76–78^

### Analytical ultracentrifugation (AUC)

Analytical ultracentrifugation has been known since the 1920s and experienced a renaissance in the 1990s, with the introduction of new instrumentation and major advances in data analysis.^79^ In its most frequently applied form, the sedimentation velocity experiment, it quantifies protein aggregation, but it is also capable of accessing stoichiometries and binding affinities of protein–protein interactions.^6,80^ In AUC, the sample is placed in a sample cell and spun in a high-speed centrifuge, which separates proteins according to their hydrodynamic radius (Fig. 3d). With UV or fluorescence detectors the spatial concentration distribution within the centrifugal sample cell can be measured over time.^81,82^

The spectroscopic signal obtained is a superposition of individual sedimenting species (Fig. 3e). The temporal and spatial evolution of the concentration distribution is then fitted with numerical solutions of the Lamm equations to extract protein concentrations and sedimentation coefficients.^83^ The sedimentation coefficients are used as a proxy for molecular size. In order to obtain accurate results, samples need purity exceeding 95%.^84^ With this level of purity, *K*_d_s in the range of 100 nM to mM are accessible, and can be extended to the pM range with dye-labeled proteins.^85–87^

For interactions in the mM range, high protein concentrations are required, which increases sample consumption.^88^ In exceptional cases it is possible to follow the time dependencies of interactions, but only for single kinetic rate constants on the order of 10^−4^ to 10^−3^ s^−1^, which matches the typical time scale of the sedimentation speed experiment.

General advantages of AUC are the large range of accessible molecular weights and the broad range of buffer conditions, such as complex formulation buffers used in drug development.^81^ However, the combination of limited molecular weight resolution and dependence on sedimentation speed means AUC is not suitable for studying the interactions of proteins with either very similar or very different molecular weights.

Among recent applications is the work of Chaturvedi *et al.*, where they developed a method to quantify macromolecular interactions at high concentrations (mM) while accounting for colloidal hydrodynamic interactions and thermodynamic non-ideality.^89^ In 2019, they applied their method to examine ultra-weak self-association of proteins.^66^ They initially investigated the monomer–dimer equilibrium of hen egg lysozyme, with its well-known ionic strength-dependent *K*_d_ (*K*_d_ = 24 mM at 300 mM NaCl, *K*_d_ > 53 mM at 100 mM NaCl). After this benchmarking step, they quantified the monomer–dimer equilibrium of chicken γS-crystallin. Fig. 3f shows sedimentation coefficient distributions *c*_NI_(*s*_0_) of chicken γS-crystallin from 15 μM to 4 mM (curves in different colors for different concentrations). The inset shows weight-average sedimentation coefficient *s*-values as a function of concentration (circles in different colors for different concentrations) and the best-fit isotherm for a monomer–dimer self-association model (red line) resulting in an estimated *K*_d_ of 27 (16–81) mM. For comparison, conventional analysis, not accounting for non-ideality, led to weight-averaged *s*_w_-values shown as crosses. In 2020, the authors’ work resulted in the publication of several protocols for quantitative analysis of protein self-association.^90^

At the other extreme of the concentration range, recent developments in fluorescence detected sedimentation velocity AUC enabled measurements at low pM concentrations and of pM *K*_d_s, of *e.g.* antibody-antigen interactions^85^ and homo-dimerization of the glutamate receptor GluA2 amino terminal domain.^86^ The recent work of Zhao *et al.* on homo-and heterodimerization of AMPA and kainate receptor ATD shows the power of AUC to access binding affinities over several orders of magnitude, from pM to μM.^91^ AUC has also been used to assess the binding affinities of the homodimer complexes of tumor suppressor neurofibromin.^92^

### Size-exclusion chromatography multi-angle light scattering (SEC-MALS)

Light scattering techniques for protein–protein interaction measurements include static (SLS) and multi-angle (MALS) light scattering, as well as dynamic light scattering (DLS). Given the broad availability of commercial SEC-MALS systems we focus herein on applications of the MALS technique. SEC-MALS applies UV absorption and multi-angle light scattering detectors to the effluent of gel filtration columns to quantify the abundance, molecular weights, stoichiometry, estimated binding affinities and aggregation of protein–protein complexes.^93–97^

Size-exclusion chromatography (SEC) makes it possible to separate a sample into its different components according to the shape and size of molecules, reducing the complexity of the sample (Fig. 3g). After the sample passed through the SEC column, the UV detector quantifies absolute concentrations, and the combination of MALS and refractive index (RI) detectors enables the online quantification of the average molecular weights of the biomolecules in the eluent.

In MALS, a monochromatic light source (*e.g.* a laser) is directed onto a sample volume, in a glass cuvette or directly within the SEC system. Scattered light can be detected at multiple angles simultaneously. The light scattering of macromolecules in solution is then used to derive their hydrodynamic radius and molecular weight distribution.^98,99^ Compared to SLS, which detects scattered light at one, fixed angle, MALS yields results with higher confidence.

In theory, the averaged scattered light intensity can be calibrated against a simple reference standard (*e.g.* toluene).In practice, however, calibration with a molecular weight standard is recommended because it allows for calibration of all detectors at the same time and can be easily repeated.^93^ After calibration of the UV, RI and MALS detectors, and measurements at different angles, an average molecular weight is obtained for biomolecules present in the eluent(Fig. 3h).^100,101^

In combination with absolute concentration measurements, the average molecular weight and the peak areas of the SEC profile are used to determine the abundance of bound and unbound species, and with that, the binding affinity of protein–protein interactions.^100^ Because of the ongoing dilution and separation within the SEC column, true equilibrium is only achieved under carefully chosen conditions, where kinetics are very rapid or very slow on the time scale of the separation.^46^ Therefore, binding affinities obtained under the assumption of fast or slow equilibration should be considered as estimates.^102^

Methods that ensure the system reaches equilibrium, such as the Hummel–Dreyer method or large-zone equilibrium gel-filtration, have received limited attention due to requiring large sample amounts.^4^ For measuring binding affinities of self-associating proteins, SEC-MALS is proposed as a simple alternative to AUC.^103,104^ In 2010, Kapoor *et al.* showed that the multi-domain calcium-binding protein Nucleobindin 1 (NUCB1) exists as a dimer and quantified the monomer–dimer dissociation constant with SEC-MALS. Fig. 3i shows measurements of NUCB1 fractions with increasing protein concentration in 50 mM Tris–HCl, pH 8.0, 150 mM NaCl. The average molecular weight of the complex at a given protein concentration was determined from a non-linear least squares fit of a collection of values determined for the apex fractions of each eluting peak. A monomer–dimer association model of the values as a function of sNUCB1 concentration yielded an apparent *K*_d_ of 0.26 ± 0.12 μM. The error bars indicate the extent of variation in molecular mass determination originating from the light scattering measurement. The molecular weight of a truncated mutant sNUCB1 (W333Ter), where the structural motif for dimerization was removed, is shown in red.

Although used in the past to quantify binding affinities of proteins^6,93^ publications from 2017 onwards suggest that SEC-MALS is now mostly used to assess purity, molecular weight, stoichiometry^105^ and aggregation. In these publications, SEC-MALS is then complemented by techniques such as SPR,^106–108^ MST,^106^ ITC,^109–111^ AUC,^92^ fluorescence spectroscopy^112^ and flow cytometry^113^ to quantify binding affinities.

## Solution-based methods

### Composition-gradient multi-angle light scattering (CG-MALS)

**Fig. 4:**
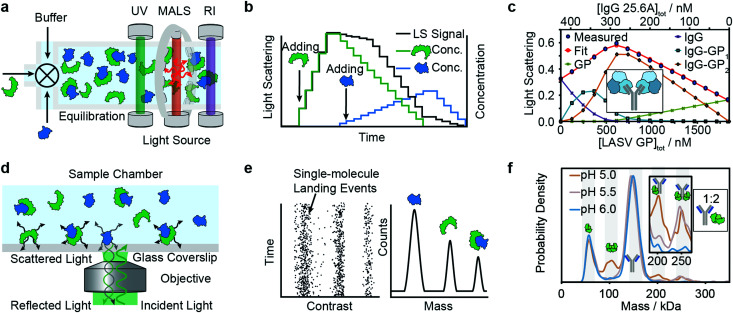
Solution-based methods for PPI quantification. Principles and experimental data of composition-gradient multi-angle light scattering (a–c) and mass photometry (d–f) are shown. Adapted from ref. 114 with permission of Elsevier (c) and from ref. 115 with permission from Wiley (f).

Composition-gradient multi-angle light scattering (CG-MALS) has an identical detection principle to SEC-MALS but allows for protein–protein interaction measurements at equilibrium. Commercially introduced in 2010, CG-MALS can measure molecular weight distributions, binding affinities (pM to mM) and kinetics (seconds to hours) for biomolecules with molecular weights ranging from 10^3^ to 10^9^ Da. The composition gradient system can mix up to 3 components in varying ratios and total concentrations before being injected into a stop-flow cell (Fig. 4a).

Like in SEC-MALS, the flow cell is monitored by a UV, RI and MALS detector to measure the total concentration and average molecular weight of the macromolecule mixture in a time-resolved manner. Data is acquired for a series of different sample concentrations or compositions (Fig. 4b). The average molecular weight, quantified with the MALS detector, serves as a measure for the stoichiometry of the complex and, most importantly, the ratio of bound and unbound species. Fitting a binding model to the average molecular weight of the specie *vs.* the absolute concentration allows extraction of the binding affinity values. Typical experiments have an unattended duration of *ca.* 3 h and software is available for analysis of the binding data.^114^

The advantages of CG-MALS are that it offers label-free measurements, requires minimal sample preparation and possesses broad buffer compatibility. However, compared to other methods, CG-MALS’ sample consumption—often several hundred μg per experiment—is relatively large. Moreover, the limited mass precision of MALS means that molecular weights often require further confirmation *via* alternative methods. The limited mass resolution makes experiments involving small molecular weight differences challenging.^98^

Alternative approaches with dynamic light scattering (DLS), instead of MALS, suggest that low sample consumption (few μl of nM to mM per data point) can be achieved.^98,116,117^ High-throughput approaches have also been described in the literature.^118^

Among recent applications are studies focused on guiding mAb discovery and formulation by quantifying self-association binding affinities and oligomerization.^119–123^ Pallesen *et al.* used CG-MALS to complement BLI studies of Ebola virus GP and antibodies.^124^ In 2019, Hastie *et al.* applied CG-MALS to confirm binding affinities of Lassa virus surface glycoprotein (GPC) and antibodies.^114^Fig. 4c shows representative CG-MALS data of LASV GPCysR4 and GPC-B IgG 25.6A.^114^ The data displayed is the light scattering intensity as a function of composition. Blue circles indicate the measured light scattering intensity for each gradient plateau; red circles indicate the fit to a model of up to two GPC monomers bound to one IgG with a *K*_d_ of 9.8 ± 1 nM. The contribution from each species (GPC, IgG and GPC–IgG complexes) to the total intensity of light scattered is shown according to the legends. Hastie *et al.* showed that the obtained *K*_d_s in the nM range were in good agreement with their BLI experiments.

These experiments show that CG-MALS can quantify binding affinities, stoichiometries and the molecular weight of biomolecules. Although measurements can be run automatically and data analysis is supported by appropriate software, future efforts could help to further improve throughput and decrease sample consumption.

### Mass photometry (MP)

Mass photometry is a label-free, optical method to quantify molecular weights of biomolecules at the single-molecule level by interferometric detection of light scattering.^125^ MP is applied for the *in vitro* quantification of molecular weight distributions, purity, aggregation, stoichiometry, binding affinities and kinetics of biomolecules and their complexes.

In MP, a sample is added to a glass substrate (*e.g.* microscope coverslip). Illumination of the glass surface by a laser generates reflected and back-scattered light from the glass–water interface, which is detected by a camera (Fig. 4j). Small scatterers (compared to the wavelength of light) landing on the glass–water interface cause a change in local refractivity and generate a light scattering signal, which is proportional to their molecular weight. This relationship allows for label-free detection and molecular weight measurement of biomolecules at the single-molecule level.

After calibration with molecular weight standards, the light scattering signals of hundreds to thousands of single biomolecules (depending on experimental conditions) can be converted into molecular weights and represented as a molecular weight distribution, similar to mass spectra (Fig. 4k). MP reports molecular weight distributions with up to 2% mass accuracy, up to 19 kDa resolution, and 1 kDa precision.^125^

These molecular weight distributions accurately measure distributions of biomolecules in bulk solution.^115^ From this, one can extract binding affinities, kinetics and stoichiometries. Extension of the accessible concentration range with a microfluidic injection system allowed for the quantification of protein–protein interactions with binding affinities from low pM to 200 nM and kinetics on the timescale of minutes to hours.

For a light scattering technique, MP possesses exceptional molecular weight resolution, which has made it possible to use MP to resolve even complex stoichiometries, such as the pH-dependent interaction of FcRN and IgG, which involves 5 co-existing species.^115^Fig. 4l shows binding affinity measurements of self-assembly of FcRn dimers and formation of IgG–FcRn complexes (1 : 2 mixture) at different pHs. With these measurements, *K*_d_ values for FcRN monomer–dimer as well as the FcRN–IgG equilibrium and their pH-dependence were obtained. Furthermore, the pH-dependent binding affinities of interactions revealed cooperativity in FcRn binding to the IgG.

Besides the high molecular weight resolution for a solution-based technique, the ability to resolve interactions of co-existing species and measuring on- and off-rates, MP has practical advantages such as low sample consumption (a few μl of nM sample per data point), compatibility with a wide range of physiological buffers, and minimal sample preparation requirements.

A disadvantage is that current MP instrumentation can only access particle concentrations below about 100 nM (nominal monomer concentrations are higher for oligomerized molecules), which also limits the accessible *K*_d_ range from low pM to few hundred nM (for monomeric species). Additionally, working with purified samples is required to remove non-specific background. The data from MP binding experiments is straightforward to analyze because relative abundances of different species can be directly extracted, but running control experiments at different dilutions and time points is advised, to confirm equilibrium conditions.

A comparison by Wu *et al.* of MP with established binding affinity measurement techniques showed that MP binding affinity data of antibody-antigen interactions was in agreement with ITC and BLI measurements and superior in terms of stoichiometry determination.^126^ Recently, MP has also been used to quantify binding affinities in the self-association of proteins (FOXP2 oligomerization,^127^ tubulin dimerization,^128^ CaMKIIα oligomerization^129^), and for qualitative assessment of affinities between serum haptoglobin protein and hemoglobin^130^ and casposase and DNA.^131^ Recently, Li *et al.* and Olerinyova *et al.* showed that MP methods can be extended to other classes of biomolecules, such as DNA^132^ and membrane proteins.^133^

Further development of MP will likely focus on improvements in molecular weight and concentration range, as well as time resolution. Given the simplicity of the injection and data acquisition procedures, implementation of fully automated and high throughput instruments seems achievable in the near future.

## Conclusions

Several characteristics are important to consider when selecting a biophysical optical method for protein–protein quantification. That is applicability to various classes of biomolecules (*e.g.* proteins, DNA, glycans, *etc.*), dynamic range in molecular weight, binding affinities and kinetics and resolution of stoichiometry. It is also important to consider requirements for immobilization, labelling, sample consumption, degree of automation, throughput and reproducibility. Although many techniques performed well in several of these areas, there was no “ideal” technique which can cover all aspects. Moreover, there remain fundamental challenges in the field, such as non-specific adsorption of biomolecules to surfaces or ensuring equilibrium conditions. In the future, we see potential for developments focusing on label-free and immobilization-free methodologies, miniaturized instrumentation and automation. Beyond accessing the unperturbed interactions, simplifying sample preparation and increasing throughput, these developments will make these methods more user-friendly, accessible and widely applied.

## Author contributions

Concept, F. S. and P. K.; methodology, F. S.; investigation, F.S.; visualization, F. S.; writing – original draft, F. S.; writing – review and editing, F. S., W. B. S. and P. K.; supervision, W. B. S. and P. K.

## Conflicts of interest

P. K. is a founder, shareholder and director to Refeyn Ltd, W. B. S. is a shareholder and consultant to Refeyn Ltd. All other authors declare no conflict of interest.

## Supplementary Material
